# Supplementary material for: Loss of CREBBP and KMT2D cooperate to accelerate lymphomagenesis and shape the lymphoma immune microenvironment
Source: Nat Commun. 2024 Apr 3;15:2879. doi: 10.1038/s41467-024-47012-1 (PMC10991284; doi:10.1038/s41467-024-47012-1)

Supplementary Figure 1

a, Odds ratio (OR) analysis of *CREBBP* and *KMT2D* mutations co-occurrence among EZB/Cluster3 DLBCL (merged DLBCL datasets) and FL_all (merged FL datasets) cohorts of patients. EZB/Cluster3 contains 319 patients, out of which 89 (27.9%) carry CREBBP mutation, 98 (30.7%) carry KMT2D mutation, 37 (11.6%) carry both mutations. FL_all contains 478 patients, out of which 250 (52.3%) carry CREBBP mutation, 305 (63.8%) carry KMT2D mutation, 185 (38.7%) carry both mutations. The p values were calculated by two-tailed Fisher's exact test.
b-c, Genotyping PCR confirming the Cre-mediated heterozygous knock out of *Crebbp* (**b**) or *Kmt2d* (**c**) in day 235 murine lymphoma samples (4 replicates per genotype). Top: PCR primer location, with LoxP sites depicted as gray triangles. Bottom: agarose gel image of PCR product.
d, Representative spleen images of mice euthanized at day 116 and 235 post BMT.
e, Representative H&E and B220 IHC images of kidney and liver sections from mice euthanized at day 116 post BMT. The scale bars represent 380 pixels.
f, Representative H&E, B220 and Ki67 IHC images of spleen sections from mice euthanized at day 235 post BMT. The scale bars represent 200 pixels.
g-h, Representative FACS plots show the gating strategy and frequency of splenic GC B cells ($B220^{+}CD38^{-}FAS^{+}$) in mice at (**g**) day 116 and (**h**) day 235 post BMT.
i, FACS analysis showing the relative abundance of splenic total B cells ($B220^{+}$) normalized to total single cells at day 116 and 235 post BMT (mean ± SD). Each dot represents a mouse (n=4 mice per genotype).
j, Stacked bar plots showing the fraction of different Ig heavy chain isotype genes expressed in day 235 murine lymphoma samples based on RNA-seq data.
Source data are provided as a Source Data file.

Supplementary Figure 1

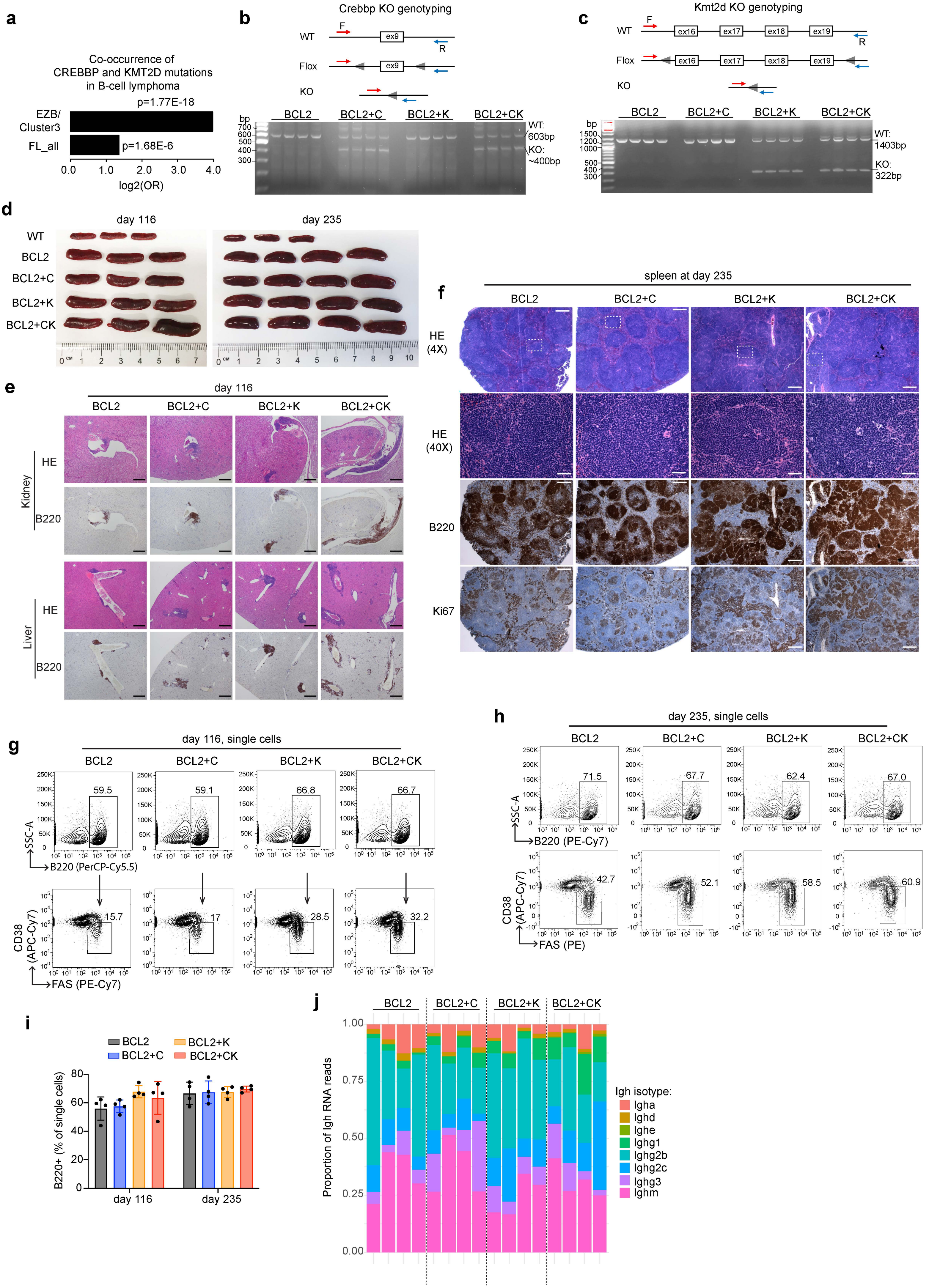

Supplementary Figure 2

a, Bar graphs depicting the number and fraction of all distinct IgH clones in each genotype at day 235 post BMT. Each bar represents a mouse (n=4 mice per genotype).
b, Pie charts showing the fractions of different IgH clonality (top) or diversity (bottom) categories in each genotype. The 25th and 75th percentiles were used as cut-off for intermediate vs low and high vs intermediate respectively.
c, Representative FACS plots show the gating strategy and relative frequency of splenic $CD4^+$ and $CD8^+$ T cells at day 116 and 235 post BMT.
d, e, g, Representative FACS plots show the gating strategy and frequency of different splenic CD8 subtypes (**d**, naïve/CM/effector CD8 at day 116, **e**, $CD44^+$ activated CD8 at day 235, **g**, $TCF1^-TOX^+$ exhausted CD8 at day 116 and 235). In panel **e**, we were unable to separate naïve, CM, and effector CD8 due to CD62L shedding after freeze-thaw cycles.
f, FACS analysis showing the relative abundance of splenic $CD44^+$ $CD8^+$ cells at day 235 (mean ± SD). Each dot represents a mouse (n=4 mice per genotype).
h-i, Representative FACS plots show the gating strategy and frequency of splenic Tfh ($CD4^+CXCR5^+PD1^+FOXP3^-$) and Tfr ($CD4^+CXCR5^+PD1^+FOXP3^+$) cells at day 116 (**h**) or day 235 (**i**) post BMT.
j-k, FACS analysis showing the relative abundance of splenic Tfh (**j**) or Tfr (**k**) normalized to $CD4^+$ cells at day 116 and 235 post BMT (mean ± SD). Each dot represents a mouse (n=4 mice per genotype).
l-m, FACS analysis showing the ratio of Tfh vs Tfr (**l**) or Tfh vs GCB (**m**).
n, Representative FACS plots show the gating strategy and frequency of splenic Treg cells ($CD4^+FOXP3^+$) at day 116 and 235 post BMT.
o, FACS analysis showing the relative abundance of splenic Treg normalized to $CD4^+$ cells at day 116 and 235 post BMT (mean ± SD). Each dot represents a mouse (n=4 mice per genotype).
P values in panels **f**, **j, k, m**, **o** were determined using ordinary one-way ANOVA followed by Tukey-Kramer's multiple comparisons test. Source data are provided as a Source Data file.

Supplementary Figure 2

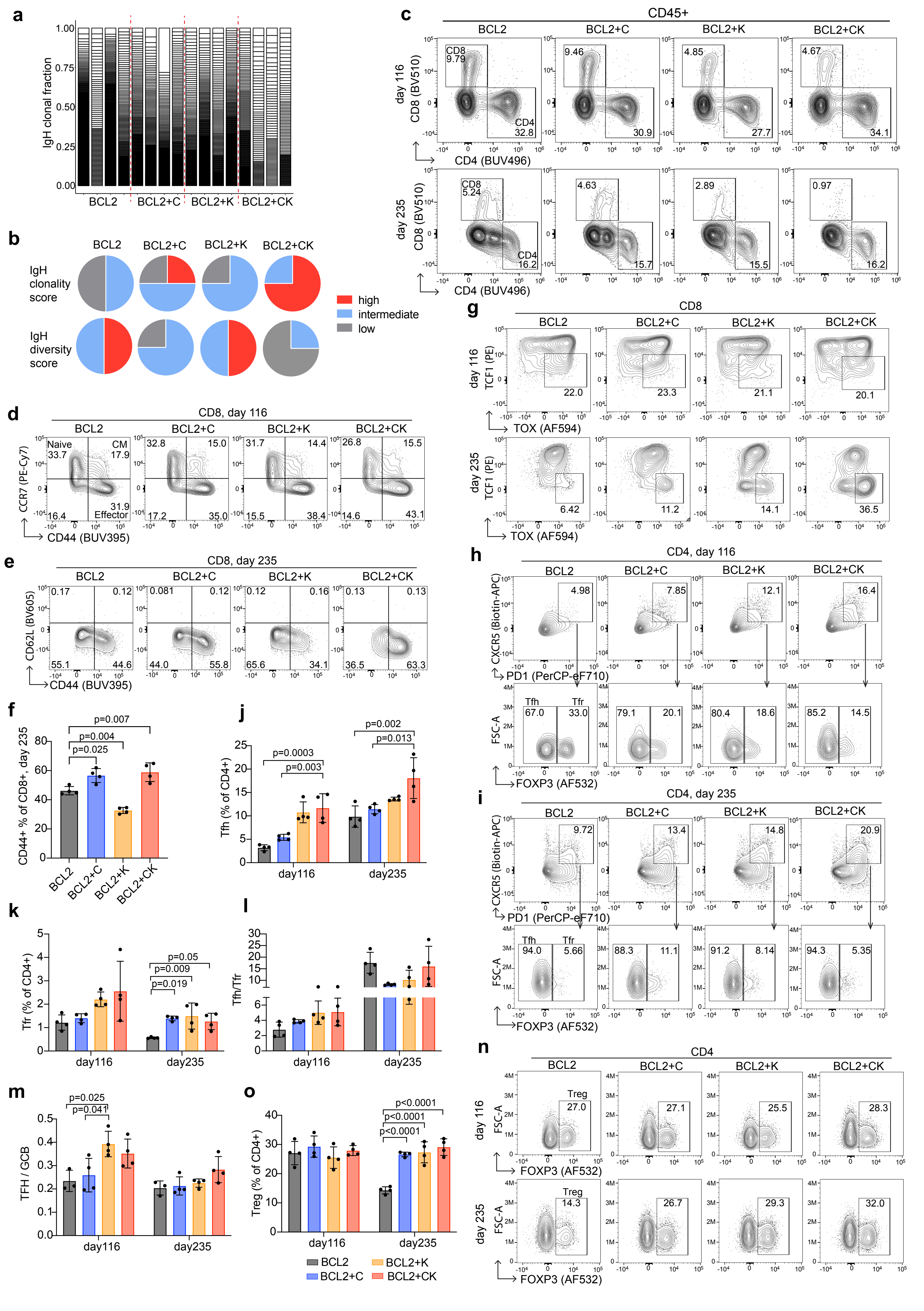

Supplementary Figure 3

a-b, Bar plots comparing the gene expression changes and showing DEGs overlap in (**a**) CB and (**b**) CC among C, K, and CK relative to WT. Orange, gray and purple colors depict upregulated, unchanged and downregulated genes as compared to WT ($|\log2FC| > 0.58$, $q < 0.01$).

c, Fuzzy c-means clustering of RNA-seq datasets identified 8 clusters (named as Traj_1 to Traj_8) with distinct trajectory patterns: line plot of standardized log2 fold-change relative to mean of WT CB. Black lines represent cluster centroids; genes are colored by the degree of cluster membership.

Supplementary Figure 3

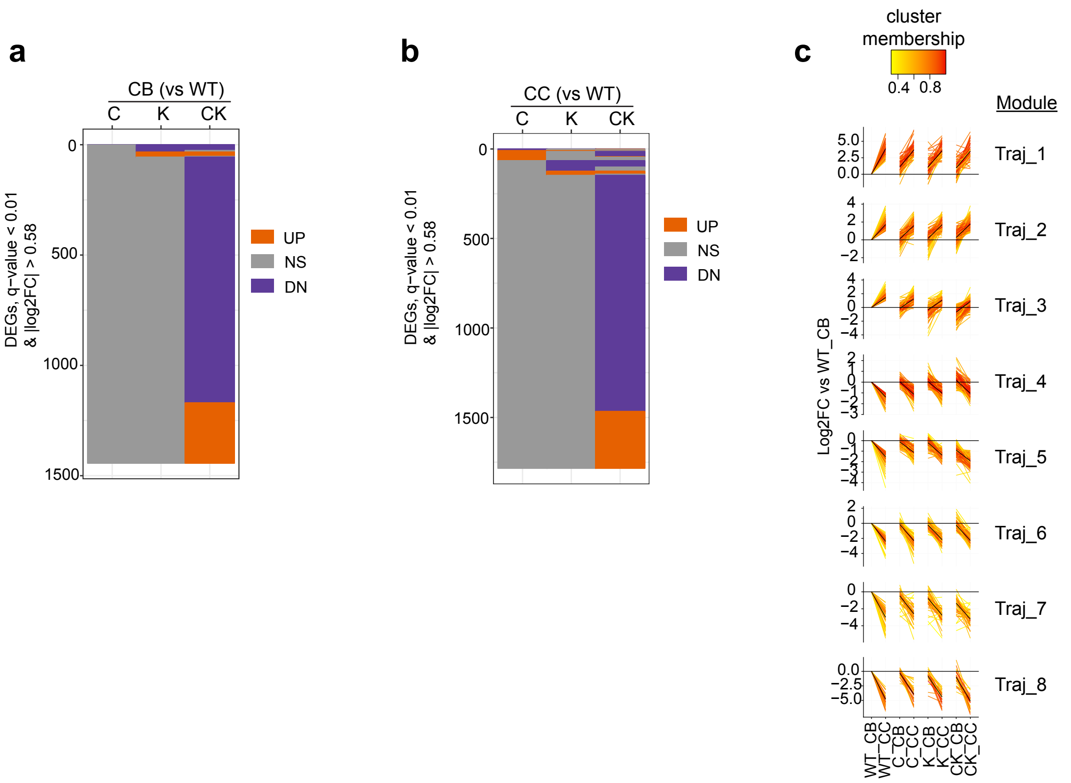

Supplementary Figure 4

a, UMAP representation of single-cell RNA-seq datasets for each genotype.
b, Dot plot showing the average expression levels and percent of cells expressing the indicated genes in different GC B cell subtypes.
c-d, Representative FACS plots show the gating strategy and relative frequency of splenic (**c**) plasma cells (PC, $B220^{lo}CD138^{+}$) and (**d**) memory B cells (MB, $CD38^{+}FAS^{-}$ $IgD^{-}CD138^{-}$) at day 10 post SRBC.
e, Experimental design for panels **f-j**.
f-h, ELISA analysis showing (**f-g**) the titers and (**h**) ratios of NP-specific high- (**f**, NP8-reacting) or low-affinity (**g**, NP28-reacting) IgG1 in serum collected at different time points post NP-OVA immunization (mean ± SD). Each dot represents a mouse (n=4 mice per genotype). P values were determined using two-tailed unpaired Student's t test.
i, Representative ELISPOT images. Each spot represents a long-lived plasma cell (LLPC) in the bone marrow capable of secreting NP8-reacting IgG1 antibody.
j, ELISPOT assay showing the number of NP8-reacting IgG1 secreting LLPCs per 3 million total BM cells for each genotype (mean ± SD). Each dot represents a mouse (n=4 mice per genotype).
k, Representative FACS plots show the gating strategy and frequency of splenic IgM^{+} and $IgG1^{+}$ GC B cells in mice at day 10 post SRBC.
l-m, FACS analysis showing the relative abundance of GC B cells (**l**, normalized to $B220^{+}$), IgM^{+} and $IgG1^{+}$ GC B cells (**m**, normalized to total GC B cells) in mice at day 10 post SRBC (mean ± SD). Each dot represents a mouse (n=5 mice per genotype). P values were determined by two-tailed unpaired Student's t test.
n, FACS data showing the frequency of GC B cells among total B cells in either control IgG or CD40L blocking antibody treated mice (mean ± SD). Each dot represents a mouse (n=5 mice per genotype). P values were determined by two-tailed unpaired Student's t test.
Source data are provided as a Source Data file.

Supplementary Figure 4

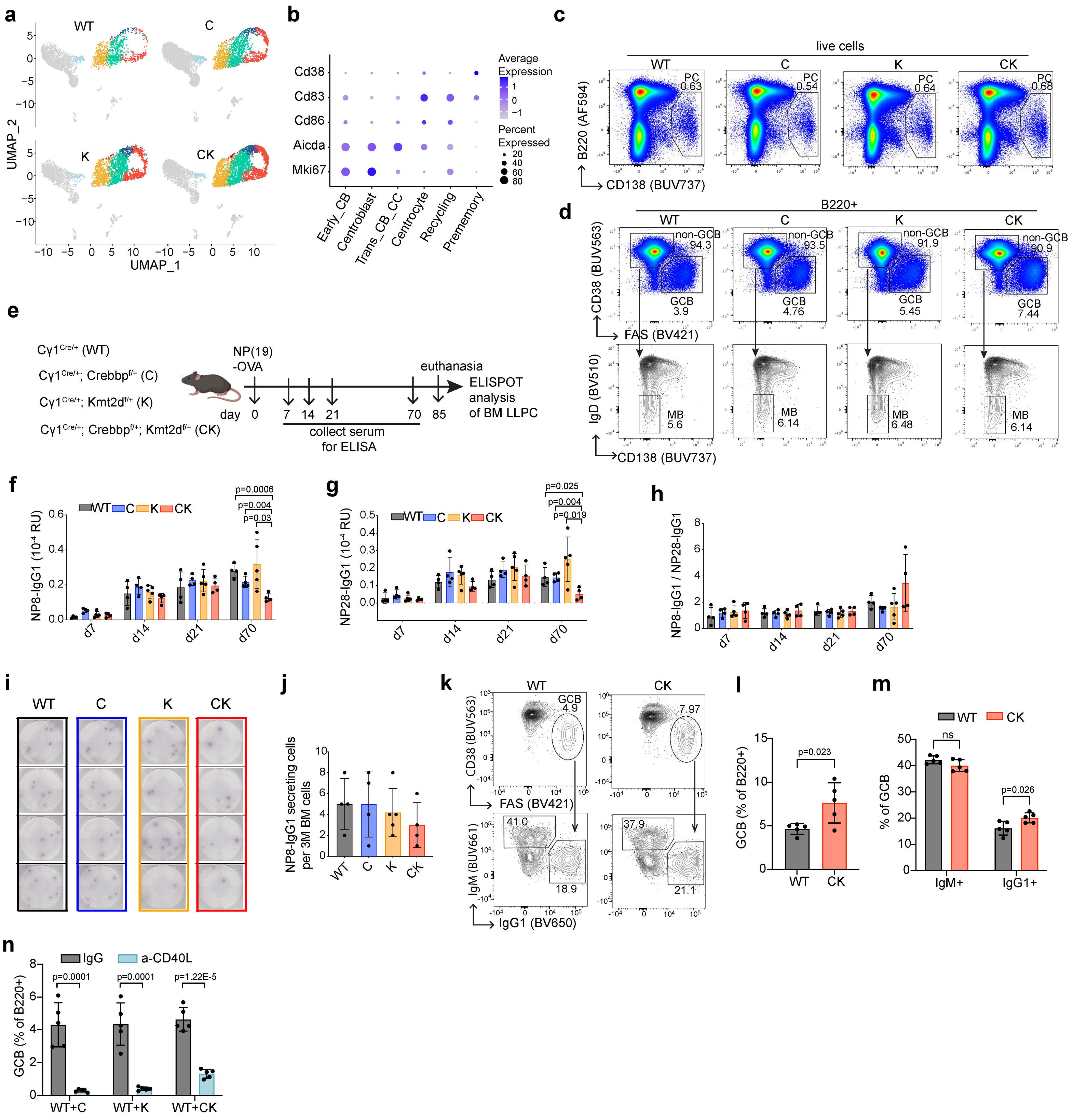

Supplementary Figure 5

a, GSEA analysis using CK_Loss target genes (annotated by GREAT) as gene set against a ranked CC RNA-seq gene list based on CK vs WT. NES, normalized enrichment score. The p value was calculated by an empirical phenotype-based permutation test. The FDR is adjusted for gene set size and multiple hypotheses testing.
b, Bar plot showing VST normalized expression values of the indicated TFs in mouse CC (mean ± SD). n=3 mice per genotype.
Source data are provided as a Source Data file.

Supplementary Figure 5

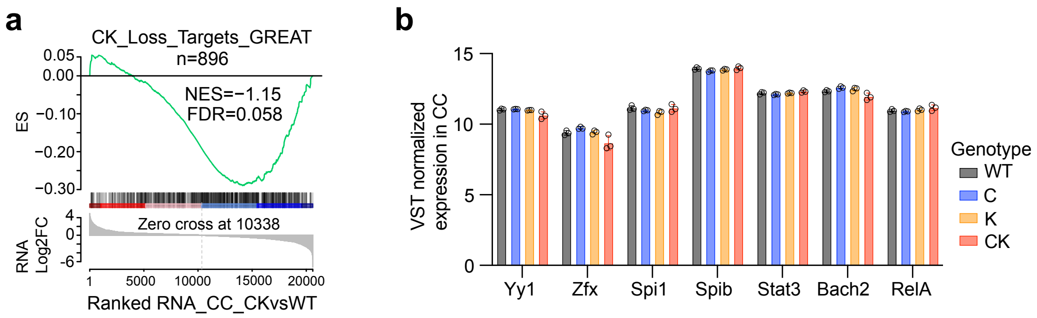

Supplementary Figure 6

a, Experimental design for generating *CREBBP*-KO and *CREBBP*-R1446C, an enzymatically dead mutant, OCI-Ly7 cell lines. ssODN: single-stranded oligodeoxynucleotide.
b, Sanger sequencing chromatogram confirming the homozygous knock out or R1446C point mutation of *CREBBP*.
c, Experimental design for generating *KMT2D*-KO OCI-Ly7 cell line.
d, Sanger sequencing chromatogram confirming the homozygous knock out of *KMT2D*.
e, Immunoblot for endogenous CREBBP and KMT2D in isogenic OCI-Ly7 cell lines using MED1 as internal loading control.
f, GSEA plot using CK vs WT upregulated genes in OCI-Ly7 as the gene set against a ranked gene list based on CK vs epigenetic WT RNA-seq datasets of human BCCA cohort GCB-DLBCL patients. NES, normalized enrichment score. The p value was calculated by an empirical phenotype-based permutation test. The FDR is adjusted for gene set size and multiple hypotheses testing.
g-h, GSEA plots using CK vs epigenetic WT (**g**) downregulated or (**h**) upregulated genes in human BCCA cohort GCB-DLBCL patients as the gene set against a ranked gene list based on CK vs WT OCI-Ly7 RNA-seq datasets. NES, normalized enrichment score. The p value was calculated by an empirical phenotype-based permutation test. The FDR is adjusted for gene set size and multiple hypotheses testing.
i, Immunoblot for H3K4me1, H3K27ac and H3 in isogenic OCI-Ly7 cells.
j-k, Relative densitometry of (**j**) H3K4me1 and (**k**) H3K27ac in panel **i**.
l, Co-IP for assessing interaction between endogenous CREBBP and KMT2D in human SUDHL4 GCB-DLBCL cell line.
m, RT-qPCR of indicated genes in isogenic OCI-Ly7 cells. qPCR signals were normalized as log2FC vs mean WT and presented as mean ± SEM. n=3 biological replicates. P values were determined using ordinary one-way ANOVA followed by Tukey-Kramer's multiple comparisons test.
n, Stacked flow cytometry histograms showing the progressive signal decrease for the indicated surface markers in C, K, and CK-deficient OCI-Ly7 cells compared to WT.
o, FACS measuring cell surface levels of the indicated markers in isogenic OCI-Ly7 cells. Mean fluorescence intensity (MFI) of each surface marker was normalized as log2FC vs mean WT and presented as mean ± SEM. n=9 biological replicates. P values were determined using ordinary one-way ANOVA followed by Tukey-Kramer's multiple comparisons test.
p-q, **t-u**, Representative FACS plots showing the gating strategy and frequency of different CD8 subtypes (**p-q**, naïve, CM, EM, and effector) or different cytokine producing CD8 cells (**t-u**, DP, IFNγ^+TNFa$^+$, DN, IFNγ^-TNFa$^-$) in the indicated co-cultures.
r, s, v, FACS analysis showing the relative abundance of total CD8 (**r**, normalized to live cells), different CD8 subtypes (**s**, normalized to total CD8), or different cytokine producing CD8 cells (**v**, normalized to total CD8) (mean ± SD). n=3 wells per co-culture. P values were determined by two-tailed unpaired Student's t test.
Source data are provided as a Source Data file.

Supplementary Figure 6

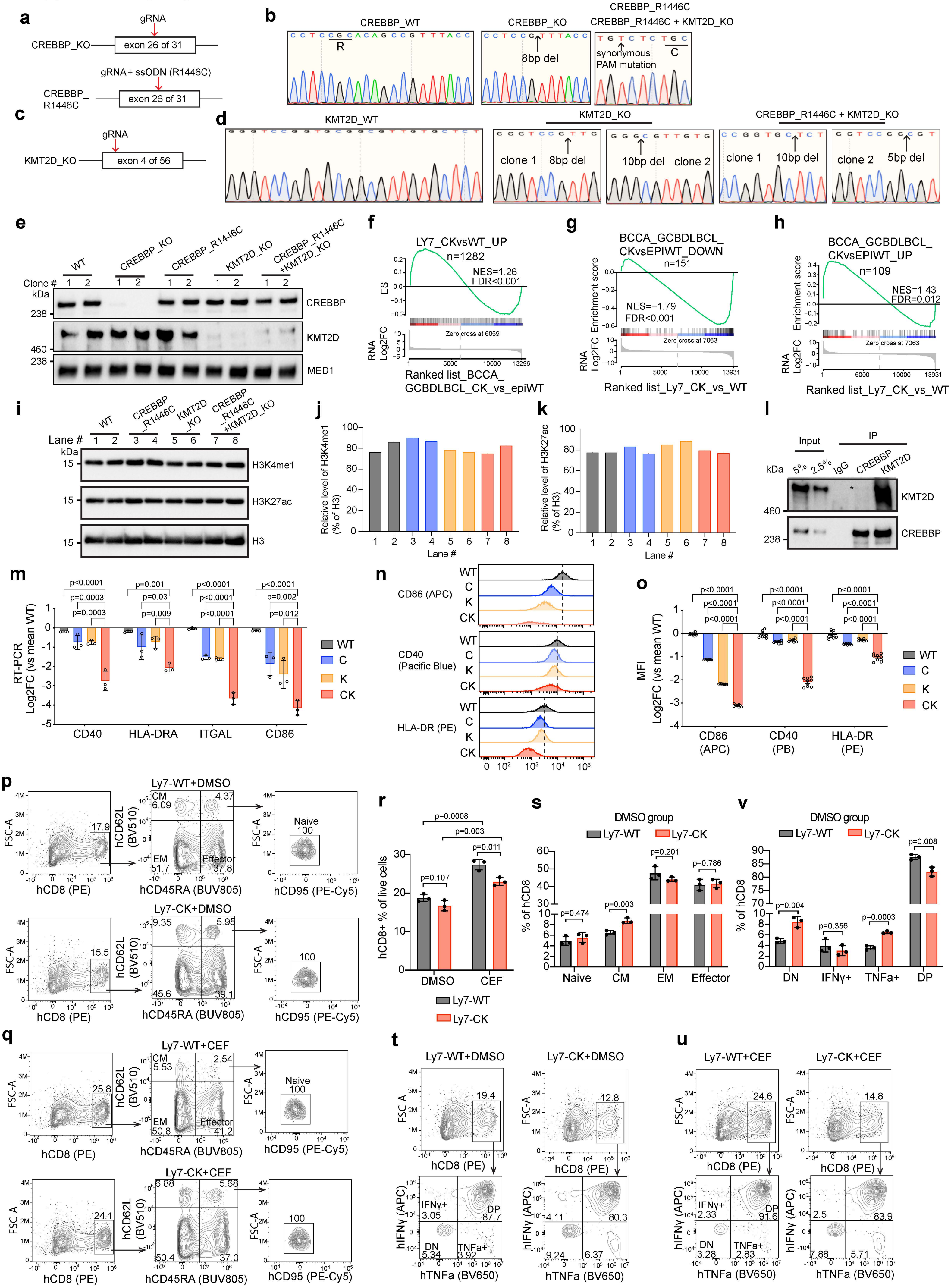

Supplementary Figure 7

a-c, Waterfall plots ranking super-enhancers (SE) in mouse GC B cells based on their accessibility change in C (**a**), K (**b**), or CK (**c**) vs WT. Constituent ATAC-seq peaks in each SE were summed before calculating the fold change. Red lines indicate absolute fold change cut off of 1.1. The numbers of opening, stable, or closing SEs are shown on top.

d-e, Dot plots comparing the TPM normalized expression levels of indicated CK repressed SE target genes among WT, C, K and CK mouse CBs (**d,** n=4/4/3/3 mice) and CCs (**e,** n=4/4/4/3 mice).

Supplementary Figure 7

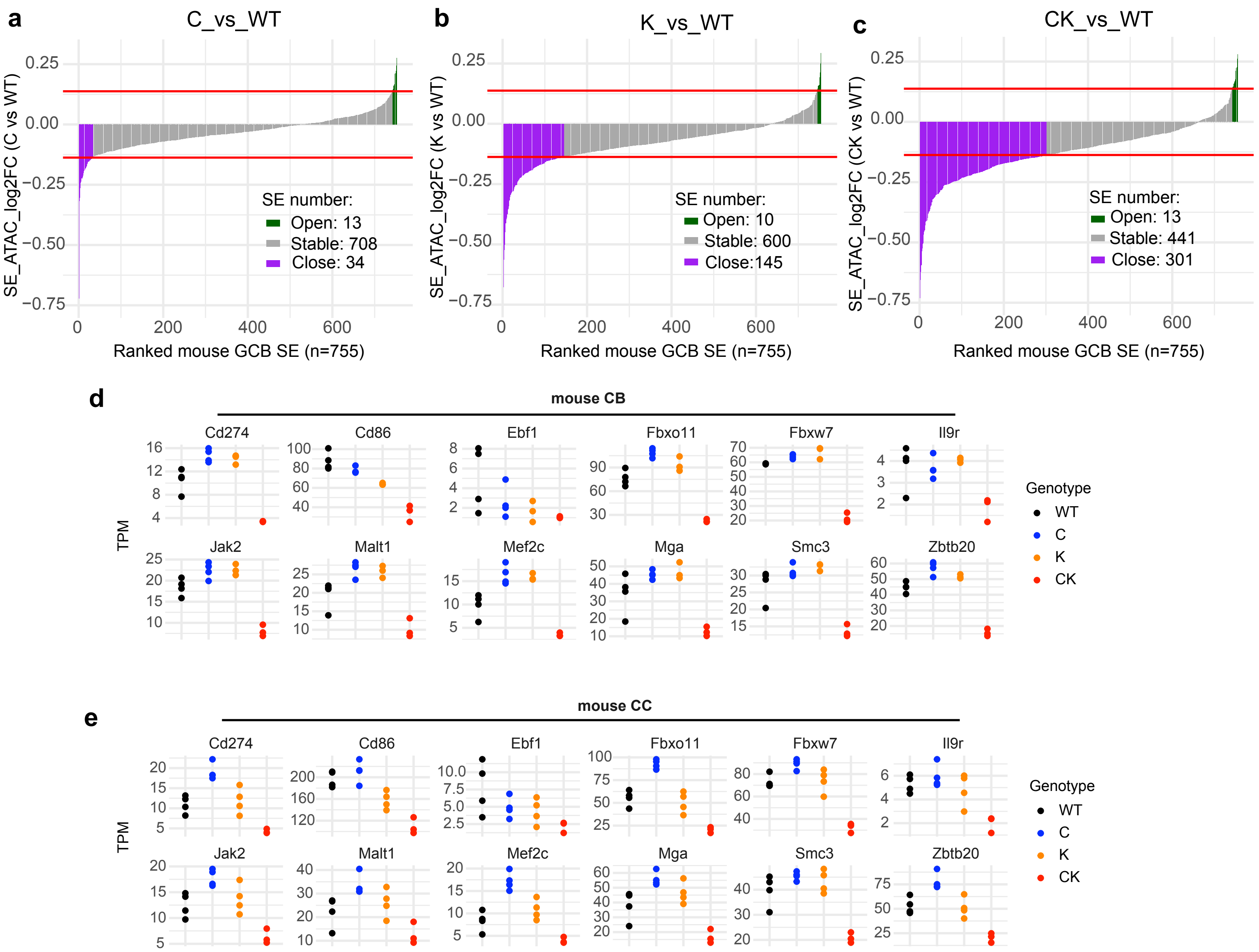

Supplementary Figure 8

a, FACS gating strategies for Fig.1d, Supplementary Fig.1i.
b, FACS gating strategies for Fig.1g-j, Supplementary Fig.2f, 2j, 2k, 2l, 2m, 2o.
c, FACS gating strategies for Fig.2b, 2d, 2h, Fig.3a, Fig.4f, 4g, Fig.5a.
d, FACS gating strategies for Supplementary Fig.6o.
e, FACS gating strategies for Fig.6p, Supplementary Fig.6r, 6s.
f, FACS gating strategies for Fig.6q, Supplementary Fig.6v.

Supplementary Figure 8

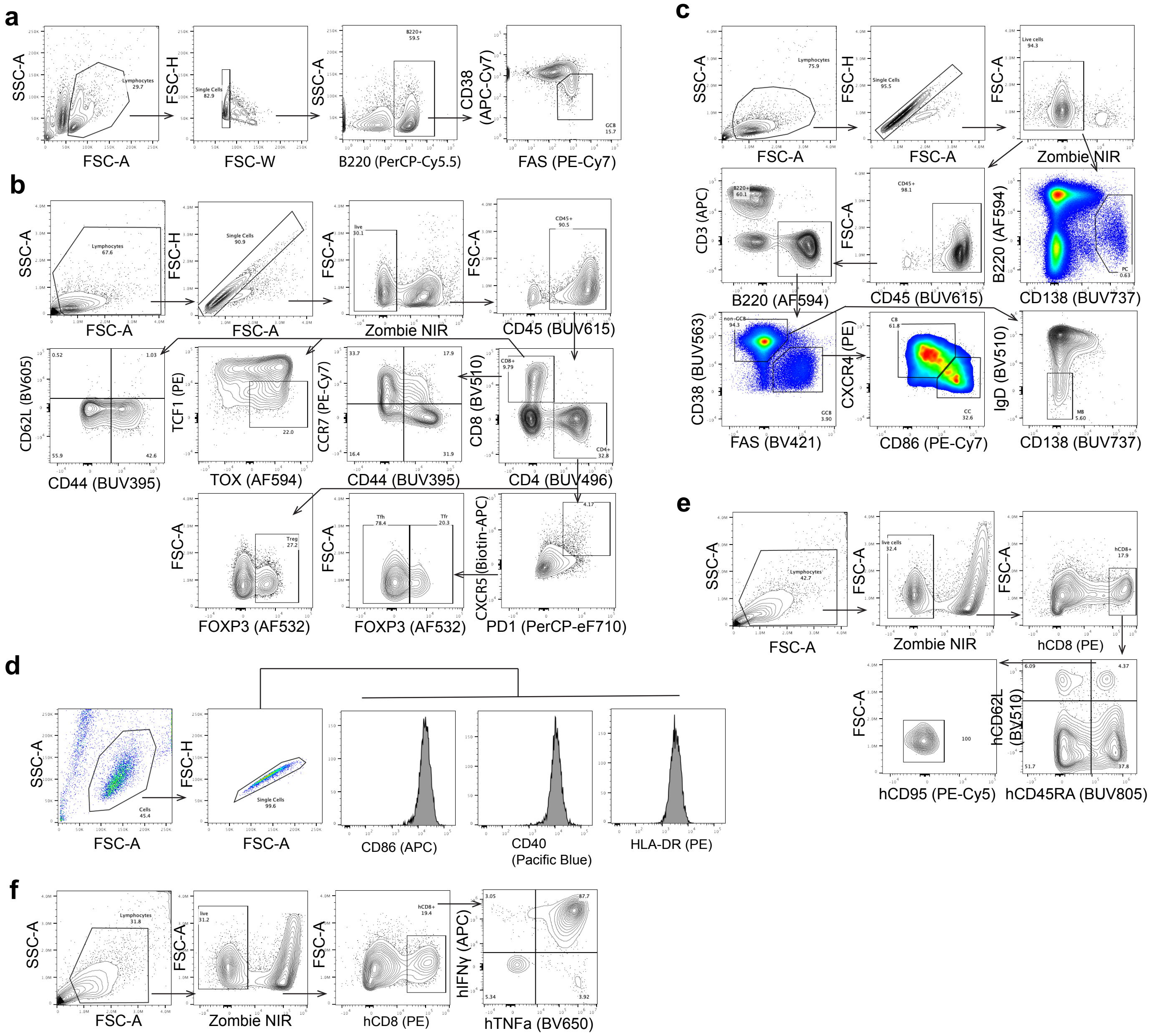

Supplement: Supplementary file 1 — Supplementary Information [file 41467_2024_47012_MOESM1_ESM.pdf]
