# Supplementary material for: Loss of CREBBP and KMT2D cooperate to accelerate lymphomagenesis and shape the lymphoma immune microenvironment
Source: Nat Commun. 2024 Apr 3;15:2879. doi: 10.1038/s41467-024-47012-1 (PMC10991284; doi:10.1038/s41467-024-47012-1)
Supplement: Supplementary file 3 — Description of Additional Supplementary Files [file 41467_2024_47012_MOESM3_ESM.pdf]

Description of Additional Supplementary Files

Supplementary Data 1: Genes carrying de novo mutations in day 235 murine lymphoma samples as revealed by whole exome sequencing.

Supplementary Data 2: DESeq2 and Fuzzy c-means clustering result of mouse CB/CC RNA-seq datasets.

Supplementary Data 3: Gene signature sets curated based on genomics data from the current study.

Supplementary Data 4: Original full names of gene signatures used in Fig.3f, 3h.

Supplementary Data 5: DESeq2 and K-means clustering result of mouse GCB ATAC-seq datasets.

Supplementary Data 6: DESeq2 and K-means clustering result of human OCI-Ly7 Cut&Run datasets.

Supplementary Data 7: Primer, gRNA and ssODN sequences used in the study.
